# Deformation engineering enables precise dispersion control in an optical microcavity

**DOI:** 10.1038/s41377-026-02297-3

**Published:** 2026-04-23

**Authors:** Julius Kullig, Jan Wiersig

**Affiliations:** Otto-von-Guericke-Universität Magdeburg, Fakultät für Naturwissenschaften, Institut für Physik, Universitätsplatz 2, 39106 Magdeburg, Germany

**Keywords:** Optics and photonics, Other photonics

## Abstract

Efficient nonlinear optical processes require the maintenance of frequency matching across a broad spectral range. Recent research demonstrates that this can be realized by tailoring the shape of an optical microdisk.

## Abstract (web summary)

Optical modes in a microcavity are tuned reliably over wide spectral ranges via boundary-shape deformations. The technique developed by Ren et al. promises seminal efficiency enhancement for nonlinear processes.

## Graphical abstract

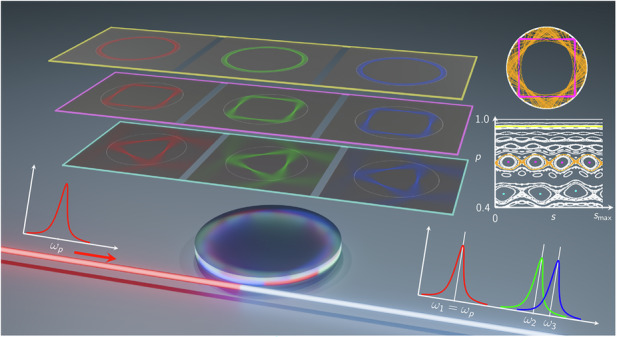

Modern nonlinear optics often struggle to sustain frequency matching when operating over extended spectral ranges. This requirement is fundamental to numerous applications, such as broadband frequency-comb generation, second-harmonic generation, and optical parametric oscillation. One well-known ansatz is to employ photonic structures known as optical microcavities. Depending on the geometry and the material dispersion, i.e., the frequency dependence of the refractive index, such a resonant structure supports a set of eigenfrequencies associated with spatial mode patterns.

Of the various types of optical microcavities, microdisks are especially noteworthy because of their conceptual simplicity, ease of fabrication, and strong light confinement. The modes in such a rotationally symmetric cavity are referred to as whispering-gallery modes (WGMs), analogous to acoustic waves that travel along the smooth wall of a circular gallery as discussed by Lord Rayleigh already in 1910; cf. ref. ^1^. Because of the rotational symmetry, these modes (for a given polarization) occur in twofold-degenerate pairs characterized by an azimuthal and a radial mode number. Fixing the radial mode number, gives a series of resonant frequencies parametrized by the azimuthal mode number. Contrary to naive expectations, the frequencies of such a series are not perfectly equidistantly spaced, even if material dispersion were negligible, which, however, is rarely the case in practical applications.

A recent article in Light: Science & Applications, authored by Prof. Yun-Feng Xiao’s team at Peking University, proposes to solve this problem by breaking the rotational symmetry of microdisk cavities through a moderate boundary deformation^2^, as illustrated in Fig. 1. Deformed microdisk cavities have been studied extensively in the field of quantum chaos, where they are called asymmetric resonant cavities. Aside from practical considerations, such as achieving directional light emission, the main focus in this field lies in understanding the classical-quantum (ray-wave) correspondence^1^. For weak to moderate deformations, the modes can be classified roughly as quasi-WGMs and islands modes. The former retain many characteristics of WGMs, e.g. the strong field concentration near the boundary, whereas the latter occur exclusively in deformed cavities and are associated with stable periodic orbits in the underlying nonlinear ray dynamics. In the ray-dynamical phase space these periodic orbits are centered inside small islands each surrounded by a sea of chaotic orbits; see Fig. 1.

**Fig. 1:**
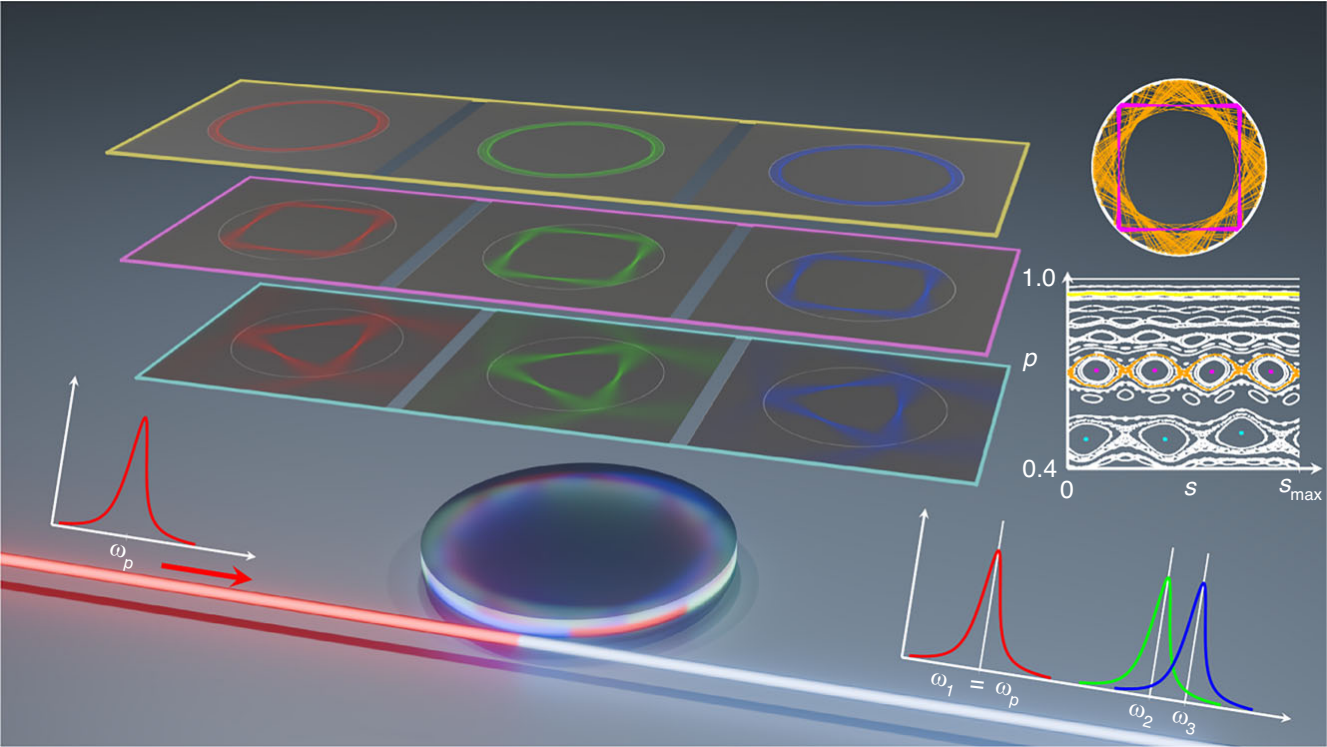
Dispersion engineering in a deformed microdisk cavity. For an effective coupling to the waveguide the excitation frequency ω_p_ has to coincide with an eigenfrequency ω_1_ of the cavity. In addition, nonlinear optical processes like second-harmonic generation or optical parametric oscillation require also frequencies that are far apart from ω_p_ to coincide with an eigenfrequency of the cavity, e.g. ω_2_ = 2ω_p_ and ω_3_ = 2ω_p_ + ∆ω. Dispersion engineering is a tool to adjust eigenfrequencies of modes over a wide spectral range. The key idea is the utilization of modes that localize along periodic ray orbits in a weakly or moderately deformed microdisk cavity. Ray dynamics in such a two-dimensional cavity is commonly analyzed through a Poincaré section of phase space, where specular reflections at the dielectric interface are described by Birkhoff coordinates, defined by the arc length s and the tangential momentum p = sin χ with χ being the angle of incidence. The phase space illustrated on the right side is mixed, meaning that regular motion, such as the stable period-4 orbit shown in pink, and chaotic motion, highlighted in orange, coexist. In a mode family corresponding to a periodic orbit, with periods 4 and 3 indicated by pink and light blue, the global dispersion coefficients are both consistent and large over a wide spectral range. Aside from such a global dispersion engineering, there is also the possibility of local dispersion engineering of quasi-whispering-gallery modes, indicated in yellow. The theory of resonance-assisted tunneling predicts how the dispersion coefficients of these mode families can be selectively tuned

Y.-F. Xiao and his team reveal that each family of island modes exhibits a markedly more uniform frequency spacing compared to the WGMs in the undeformed disk. This results from the suppression of geometry-induced dispersion by the wavelength-independent periodic ray orbits. This observation is the basis of a new highly-effective tool for global dispersion engineering in optical microcavities.

The authors also explore the mechanism of resonance-assisted tunneling (RAT), which facilitates coupling between quasi-WGMs possessing different mode numbers; see Fig. 1. RAT is a well-studied phenomenon in quantum chaos, which refers to the enhancement of tunneling rates due to island chains around periodic orbits in phase space. For asymmetric resonant cavities, RAT is relevant in the regime of weak to moderate deformation^3,4^. Y.-F. Xiao and coworkers demonstrate that RAT enables local dispersion engineering through avoided resonance crossings. Importantly, undesired couplings can be eliminated according to RAT’s selection rules.

The authors provide numerical evidence for the feasibility and usefulness of their dispersion engineering schemes by applying them to two important problems of nonlinear optics. Using local dispersion engineering, they predict for a LiNbO_3_ cavity a dramatic boost in the efficiency of second-harmonic generation: above seven orders of magnitude compared to the undeformed microdisk. Moreover, employing global dispersion engineering, the authors anticipate a remarkable total efficiency for a third-order optical parametric oscillation in Si_3_N_4_ cavity of above 55% for blue and violet light with a frequency separation exceeding 180 THz. In addition to higher conversion efficiency, here, the deformed cavity increases the waveguide-cavity coupling rate by two orders of magnitude, thereby facilitating signal extraction.

There are other approaches for dispersion engineering in optical cavities, such as employing high-index-contrast subwavelength gratings in vertical cavities^5^, implementing concentric shell structures in spherical cavities^6^, designing crystalline cavities with vertical geometries using precision machining techniques^7^, and tailoring dispersion via waveguide modification and the addition of coatings^8^. All of these approaches are based on symmetric cavities, with the notable exception of microring cavities with a modulated inner boundary reported in ref. ^9^. However, none of these studies connects so nicely different branches of physics, such as quantum chaos, nonlinear ray dynamics, and nonlinear optics.

Although the theory is convincing, it still needs to be confirmed experimentally. Given that the dispersion-engineering approaches are quite general, multiple material platforms could be employed for such studies. In this regard, it would also be interesting to explore anisotropic materials and examine how the observed effects depend on polarization. Another interesting direction arise from the generically non-Hermitian nature of optical microcavities. Here, in addition to dispersion engineering also a boundary-deformation driven dissipation engineering might be possible to enhance non-reciprocal frequency conversion in microcavities^10^. Moreover, fully asymmetric boundary deformations, which eliminate all mirror-reflection symmetries, provide the possibility to additionally adjust the overlap and non-orthogonality of modes^11^, which might further enhance the efficiency of nonlinear optical processes. Overall, this work demonstrates the power of interdisciplinary approaches, showing how merging concepts from various fields of basic physics contributes to solving practical problems.
